# Case report: Unilateral papilledema in a dog with a large suprasellar mass and suspected intracranial hypertension: insights from funduscopy, optical coherence tomography, and magnetic resonance imaging

**DOI:** 10.3389/fvets.2024.1372802

**Published:** 2024-06-12

**Authors:** Heejong Cho, Mihyun Choi, Sukjong Yoo, Manbok Jeong, Shin Ae Park

**Affiliations:** ^1^Yoolim Animal Eye Clinic, Seoul, Republic of Korea; ^2^Bon Animal Medical Center, Suwon, Republic of Korea; ^3^Department of Veterinary Clinical Sciences, College of Veterinary Medicine, Purdue University, West Lafayette, IN, United States

**Keywords:** brain tumors, Bruch’s membrane, canine, optic disk edema, optic nerve head, sellar mass

## Abstract

A spayed, 8-year-old female Poodle, weighing 5.7 kg, was presented with the chief complaint of vision impairment. Vision assessment, including pupillary light reflexes, menace response, dazzle reflex, and maze navigation in photopic and scotopic circumstances, revealed a negative response in both eyes except for positive direct pupillary light reflex in the right eye and positive consensual pupillary light reflex from the right eye to the left eye. Systemic evaluation, including neurologic status, blood profile, and thoracic radiographs, did not reveal any abnormalities. Complete ophthalmic examinations, ocular ultrasonography, and electroretinography did not identify a cause of blindness. Upon funduscopy, the left eye exhibited an increased optic disk diameter, blurred optic disk borders, and loss of the physiologic pit, as well as an increase in vascular tortuosity. In the right eye, there were multifocal depigmented areas in the non-tapetal fundus and several pigmented spots surrounded by a region of dull tapetal reflection in the tapetal fundus. The optical coherence tomography revealed severe anterior deformation of the optic nerve head and Bruch’s membrane in the peripapillary region of the left eye. Magnetic resonance imaging revealed an irregular, broad-based suprasellar mass, with features suggestive of intracranial hypertension, including dorsal displacement of third ventricles, a rightward shift of the falx cerebri, *trans*-tentorial herniation, perilesional edema, flattening/protrusion of the posterior sclera, and lager optic nerve sheath diameter in left side than right side. This is the first comprehensive report that describes unilateral papilledema in a dog with a brain tumor, using advanced ophthalmic and neuro-imaging modalities.

## 1 Introduction

‘Papilledema’ is a universally recognized term describing optic disk edema that arises from elevated intracranial pressure (ICP) (1, 2). Elevated ICP precipitates an increase in cerebrospinal fluid (CSF) pressure in the intracranial and orbital subarachnoid space, consequently changing the translaminar pressure gradient. This change in pressure impairs axoplasmic flow, ultimately leading to swelling in the axons and disk (1, 3). In humans, idiopathic intracranial hypertension (ICH) is known to be the most common cause of papilledema (3). Furthermore, papilledema can arise from various etiologies, including space-occupying lesions, excessive CSF production, diminished CSF outflow, compromised cerebral venous drainage, and reduced size of the cranial cavity (3). Among human patients with brain tumors, papilledema is observed in up to 15%, although the incidence can vary with the tumor type (3, 4). Reports of papilledema are sparse in the veterinary literature. The limited available studies indicate that papilledema is predominantly associated with brain tumors, with its prevalence varying and appearing in up to 50% of dogs affected by such tumors (5–8). In humans, differentiating true papilledema from a swollen optic disk due to other causes, such as pseudopapilledema, has long posed a significant challenge (3). The Frisén scale is a standardized staging system used to diagnose and classify papilledema in humans, based on ophthalmoscopic signs (9, 10). However, the inherent limitations of an ordinal, non-continuous system have compelled researchers to seek a more objective and accurate diagnostic method, such as optical coherence tomography (OCT) (3, 10, 11).

Current veterinary literature on papilledema lacks objective criteria for diagnosis and concrete evidence of ICP elevation. The purpose of this case report was to describe the funduscopic and OCT findings in the retina and optic disk of a canine patient with a large suprasellar mass and presumed ICH, diagnosed based on magnetic resonance imaging (MRI) findings.

## 2 Case description

### 2.1 Presentation

An 8-year-old spayed female Poodle, weighing 5.7 kg, was referred for evaluation of visual impairment. The owner found mydriasis in the left eye 6 weeks prior, followed by the right eye 2 weeks later, resulting in blindness in both eyes.

### 2.2 Complete ophthalmic examination and ocular ultrasonography

All procedures were carried out for diagnostic purposes after obtaining the owner’s informed consent. Upon presentation, the vision assessment, which included pupillary light reflexes (PLRs), menace response, dazzle reflex, and maze navigation in photopic and scotopic circumstances, revealed a negative response for all tests in both eyes except for positive direct PLRs in the right eye and positive consensual PLRs from the right eye to the left eye. Corneal, palpebral, and oculo-vestibular reflexes were normal in both eyes. The intraocular pressure (TonoVet^®^, ICare Finland Oy) was 14 mmHg in the right eye and 15 mmHg in the left eye. Slit-lamp biomicroscopy (LS-5, Sunkingdom Medical Instrument Co., Ltd.) and ocular ultrasonography (LOGIQ e, GE Healthcare) with 18 MHz transducer revealed no intraocular abnormalities that could cause visual impairment.

### 2.3 Funduscopic examination

**Figure 1:**
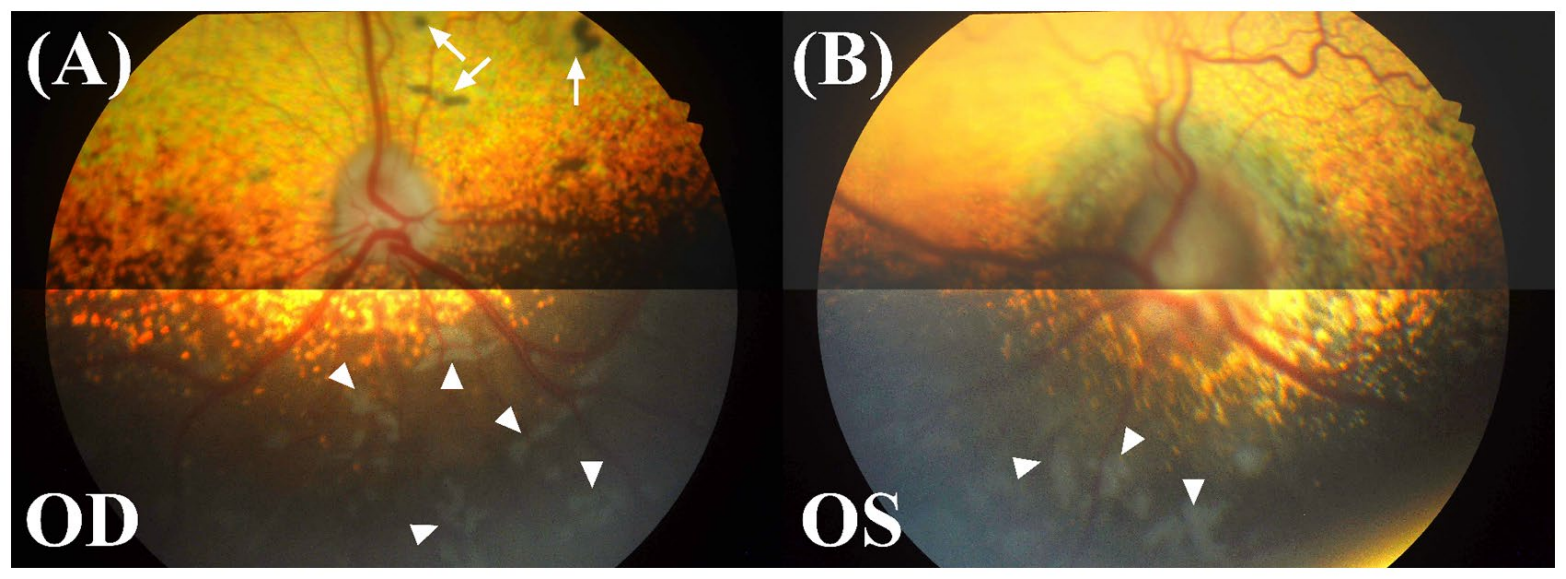
Fundus photographs of both eyes. OD, oculus dexter or right eye; OS, oculus sinister or left eye. **(A)** Multifocal depigmented areas (white arrowheads) in the non-tapetal fundus and several pigmented spots (white arrow) in the tapetal fundus were shown in the right eye. The features of the retinal vasculature, tapetal reflectivity, and optic disk all appeared to be within the normal variation. **(B)** In the left eye, an increase in the optic disk diameter, blurring of the disk margin, a change in the disk color to dark grey, multifocal depigmented areas (white arrowheads), and an increase in vascular tortuosity were observed.

Funduscopic changes were evaluated using binocular indirect ophthalmoscopy (Vantage Plus, Keeler Instruments Inc.) with a 20-diopter lens and digital fundus photographs (KOWA VX-10α, Kowa Company, Ltd.). In the right eye, multifocal depigmented areas were observed in the non-tapetal fundus, and several pigmented spots, some of which were surrounded by a region of dull tapetal reflection with blurry borders were seen in the tapetal fundus. Other characteristics, such as vascular tortuosity and diameter, overall tapetal reflectivity, and the shape of the optic disk appeared normal (Figure 1A). In the left eye, the non-tapetal fundus also had multifocal depigmented areas. The left retinal veins showed increased vascular tortuosity. The optic disk was larger compared to the right eye, with noticeable swelling, blurred margins, and no clear optic cup or physiologic pit. Additionally, the peripapillary retina presented a darker hue and appeared blurred (Figure 1B).

### 2.4 Systemic evaluation

Briefly, mental status, body posture, gait, and proprioception were normal. Blood tests, including complete blood count, serum chemistry and electrolyte, and thoracic radiographs revealed no abnormalities.

### 2.5 Optical coherence tomography

A spectral-domain OCT device (iVue 100, Optovue Inc.) was utilized to obtain cross-sectional retinal images surrounding the optic nerve head (ONH) without general anesthesia or sedation. We randomly took multiple shots to obtain high-quality photos covering a broad area from the superior to inferior retinas. In the superior retina, the thicknesses of the inner retinal layers, including the retinal nerve fiber layer (RNFL) and ganglion cell complex (GCC), appeared thinner in the left eye compared to the right eye. In the inferior retina, the thicknesses of all retinal layers appeared thinner in the left eye compared to the right eye. However, the small sample size and inconsistency of measurements in some locations, attributed to low image quality, preclude drawing statistical conclusions.

**Figure 2:**
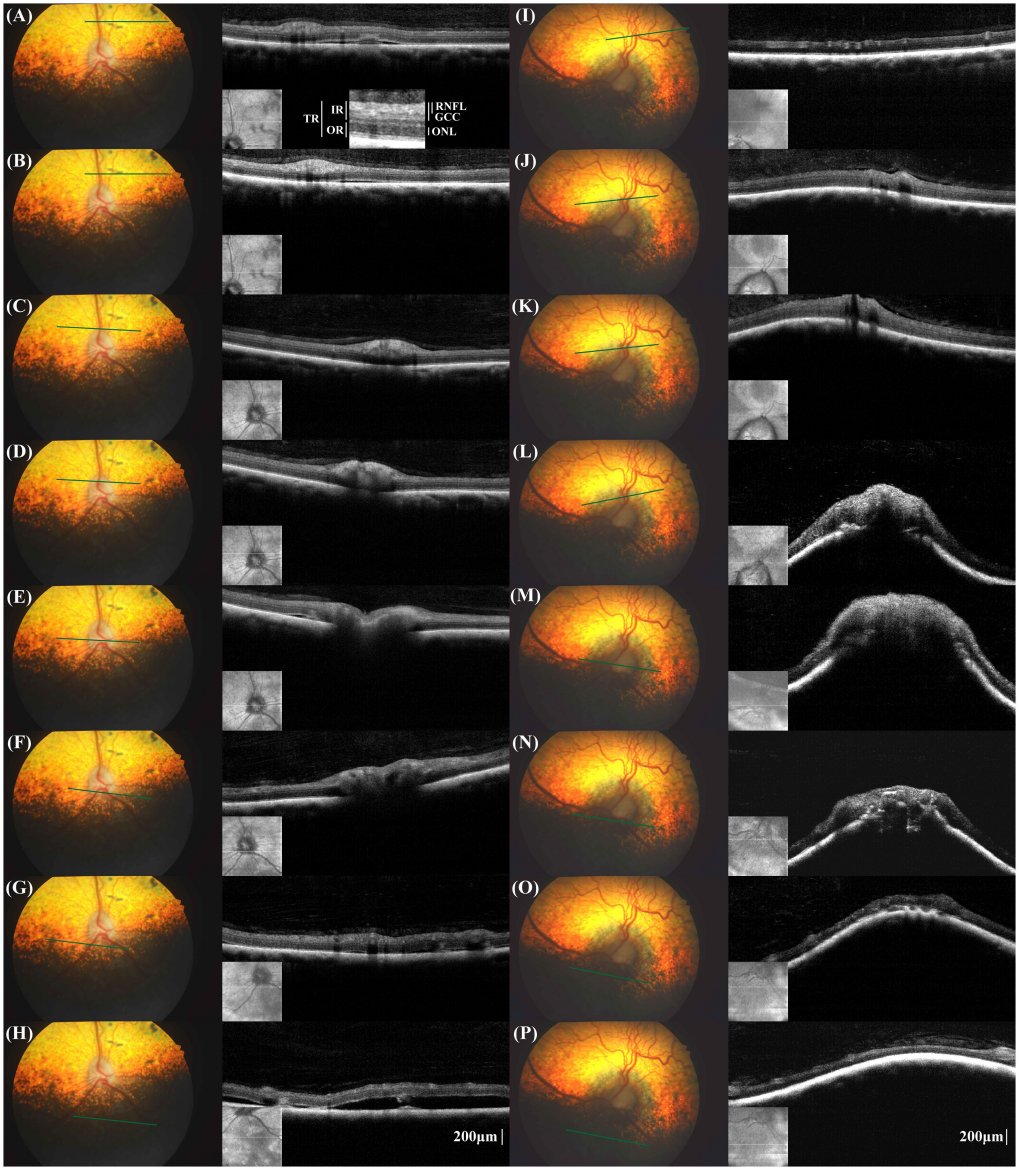
Fundus photographs and cross-sectional retinal optical coherence tomography (OCT) images in the corresponding location of the right eye **(A–H)** and left eye **(I–P)**. The measurement locations for retinal layer thickness and the designations of the measured retinal layers are schematically represented **(A)**. TR, total retina; RNFL, retinal nerve fiber layer; GCC, ganglion cell complex; IR, inner retina; ONL, outer nuclear layer; OR, outer retina. In the right eye, multifocal pigmented lesions were noted in fundus photographs, aligning with the areas of retinal detachments in the OCT images **(A,B)**. In the peripapillary region, small retinal detachment was noted despite any lesion not being shown in fundus photograph. Small retinal detachment in the non-tapetal fundus, the OCT image displayed extensive retinal detachment, exceeding the areas of the depigmented lesions seen on funduscopy **(H)**. The configuration of Bruch’s membrane layer in the peripapillary region was flat to V-shaped. In the left eye, the individual layers were not easily distinguishable, especially in the peripapillary region. A severe anterior displacement of the optic nerve head and peripapillary Bruch’s membrane was evident **(I–P)**.

The right eye showed multiple small retinal detachments, each approximately 0.5–2 mm in width, in the superior retina and peripapillary regions (Figures 2A–G). However, in the inferior retina, an extensive retinal detachment measuring over 6 mm × 6 mm was noted (Figure 2H). In the left eye, severe anterior deformation of the ONH and peripapillary Bruch’s membrane (pBM) was evident (Figures 2I–P). The left eye had two localized areas of retinal detachment in the inferior retina, each approximately 2 mm wide.

### 2.6 Electroretinography

After 20 min of dark adaptation, an electroretinogram in response to a single, bright stimulus of 3 cd s/m^2^ was recorded (LE-1000, Tomey) under sedation with intravenous medetomidine hydrochloride at a dose of 10 μg/kg (Domitor, Pfizer) in accordance with the standardized guidelines (12). Electroretinography showed normal a-and b-waves in both eyes. However, the left eye had slightly decreased electroretinography a- and b-wave amplitudes compared to the right eye (a-wave = 84.50 μV, b-wave = 188.50 μV in the left eye; a-wave = 139.00 μV, b-wave = 225.00 μV in the right eye). The above tests did not conclusively determine the cause of the visual impairment, so a magnetic resonance imaging (MRI) scan of the brain was pursued.

### 2.7 Magnetic resonance imaging

**Figure 3:**
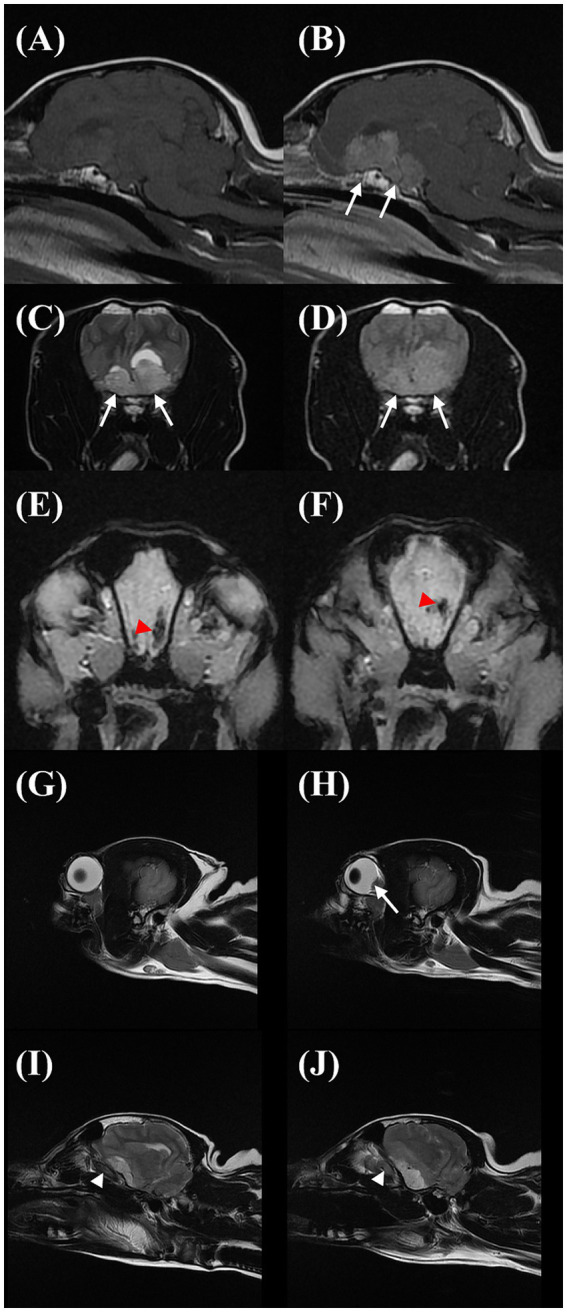
Sagittal T1W precontrast **(A)**, T1W postcontrast **(B)**, transverse T2W **(C)**, fluid-attenuated inversion recovery (FLAIR) **(D)**, and T2* **(E,F)** images showing the suprasellar mass (arrows). The area within the mass was T2W and FLAIR hyperintense to the grey matter on the T2W and FLAIR, iso to hypointense on the T1W, and moderate contrast-enhancing. Additionally, a signal void artifact (red arrowhead) was shown in the T2* sequence at the cranial margin of the mass. Sagittal T2W images on magnetic resonance imaging of the eyeball in the right eye **(G)** and left eye **(H)** and optic nerve sheath on the right side **(I)** and left side **(J)**. On the left side, there is a flattening/protrusion of the posterior sclera (arrow) and a slight increase in the optic nerve sheath diameter (white arrowhead) compared to the right side.

The MRI of the brain was performed using a 1.5 Tesla knee coil (Signa HDxt MRI scanner, GE Healthcare) under general anesthesia. The protocol included standard sequences used for evaluating the brain: sagittal and transverse T2-weighted (T2W) turbo spin echo, sagittal and transverse T1-weighted (T1W) spin echo, transverse T2-weighted fluid-attenuated inversion recovery (T2W FLAIR), transverse T2-weighted spoiled gradient echo (T2*), and transverse diffusion-weighted imaging with *b* = 1,000 s/mm^2^. An apparent diffusion coefficient map was also calculated. Additionally, the transverse, sagittal T1W, and dorsal T1 fat saturation series were acquired following gadolinium contrast administration. The brain MRI revealed an irregular, broad-based suprasellar mass measuring a minimum of 2.68 cm in length, 1.42 cm in height, and 2.34 cm in width (Figures 3A,B). The mass was heterogeneously hyperintense to the gray matter on the T2W, FLAIR, and T2* images, isointense to hypointense on the T1W images, and exhibited moderate contrast enhancement (Figure 3B). The mass was continuous with the anterior aspect of the pituitary gland, but the posterior aspect of the pituitary gland displayed normal high signal intensity on the T1W images. The mass displaced the third ventricle dorsally and caused a mild rightward shift of the falx cerebri (Figures 3C,D), and concurrent transtentorial brain herniation was also observed. A focal, well-defined area was detectable surrounding the mass parenchyma. In the most rostral left side aspect of the mass, it was hypointense to the grey matter on T2W and T1W, hypointense on FLAIR, and moderately contrast-enhancing. Additionally, a signal void artifact was visible in the T2* sequence, indicative of hemorrhage (Figures 3E,F). There was mild hyperintensity on the FLAIR images around the peripheral margins of the mass, possibly representing interstitial edema. Flattening of the posterior sclera was observed (Figures 3G,H). Additionally, there was a mild increase in the optic nerve sheath (ONS) diameter on the left side compared to the right side (right: 2.4 mm, left: 3.0 mm; Figures 3I,J). The tentative diagnosis was a large, broad-based suprasellar mass with elevated ICP and brain hemorrhage. The owner refused treatment and follow-up assessment. Follow-up contact via telephone with the owner after 1 year indicated that the dog was still blind but alive without other neurological signs.

## 3 Discussion

We delineated both the qualitative and quantitative changes of the ONH and peripapillary retinal structure in a dog with a brain tumor located in the suprasellar region. Our findings described above could be ascribed to papilledema, which was strongly supported by the neuroimaging evidence indicative of ICH.

In clinical practice, if papilledema is suspected in humans, ICP is typically assessed through lumbar puncture-measured opening pressure (3). While direct ICP measurement stands as the gold standard for accuracy and reliability, its invasive nature and high cost have spurred the exploration of non-invasive indirect modalities such as MRI, angiography, and ultrasonography to estimate ICP in human and veterinary medicine (3, 7, 8, 13, 14). Previous studies showed that MRI features such as transtentorial, foramen magnum, or subfalcine herniations, caudal displacement of the lamina quadrigemina, third ventricular compression, perilesional edema, falx shift, flattening/protrusion of the posterior sclera, and increase in the ONS diameter were significantly associated with ICH in dogs (7, 8, 13). Although ICP was not directly measured, six of the aforementioned hallmark features of presumptive ICH on MRI were identified in our case. Thus, we could reasonably speculate that the patient was indeed experiencing an elevation in ICP.

As an alternative to the fundus photograph-based Frisén scale, the utility of OCT is demonstrated in precisely differentiating papilledema from other forms of optic disk swelling, such as pseudopapilledema, in humans. OCT characteristics noted frequently included RNFL thickening, ONH volume expansion, and anterior displacement of the pBM (10, 11, 15–17). In the early stage of human papilledema, the ganglion cell layer or complex remains relatively normal (16, 17). However, when the disease advances, axonal loss leads to a decrease in the RNFL and GCC thicknesses. It is important to note that these changes do not occur in pseudopapilledema (11, 16, 17). In our case report, the GCC and RNFL thickness appeared thinner in the left eye. Given that the patient lost vision in the left eye 6 weeks prior and the brain tumor invaded the optic chiasm, the thinning of the RNFL and GCC might be due to neuronal loss from chronic papilledema and/or optic nerve damage.

In the peripapillary region, the RNFL was not distinctly separated from the adjacent layers. In humans, commercial OCT devices often fail to differentiate the RNFL in severe papilledema, which is hypothesized to be caused by the disorganization of the retinal layer due to retinal edema (11, 16). Conversely, optic disk edema does not develop in cases of severe optic atrophy where no intact axons remain within the ONH (1, 4). In fact, the precise RNFL delineation on OCT images presents a considerable challenge even in normal dogs (18), and the presence of retinal edema may have further exacerbated the difficulties.

A disparity in the ONS diameter, which is more enlarged on the left side compared to the right, lends further credence to our postulation that this case exhibited unilateral or asymmetric papilledema. Prior research investigating the relationship of the variation in the ONS diameter with ICH in dogs has indicated no difference in the ONS diameter between the right and left sides under normal and elevated ICP (13, 14). On the other hand, a series of experiments in rhesus monkeys by Hayreh (1) found that when elevating ICP experimentally using a balloon embedded in the cranium to mimic space-occupying lesions, the optic disk changes usually first appeared on the side of the balloon insertion. Furthermore, these changes were more pronounced on that side, especially in the later stages (1). In humans, papilledema is usually bilateral in patients with idiopathic ICH, and an atypical presentation of asymmetric or unilateral edema is observed only in 4–10% of cases (19). It is believed that a wide variation in the extent of communication between the subarachnoid spaces of the cranial cavity and the sheath in the optic canal region or compartmentation of the subarachnoid space of the ONS is a major factor contributing to asymmetric optic disk edema, even when increases in the CSF pressure are similar in both sides (1, 3).

In veterinary medicine, pseudopapilledema and optic neuritis are the main conditions that must be differentiated from papilledema (20). In dogs, a pseudopapilledema is characterized by prominent axonal myelination anterior to the lamina cribrosa, a process not linked to any pathological condition (20). This contrasts with human pseudopapilledema, predominantly caused by drusen formation, which sometimes leads to slow progressive visual field defects (21). Qualitative changes in the ONH and peripapillary retina, including anterior deformation of the pBM, peripapillary wrinkles, retinal folds and creases, and choroidal folds, also aid in diagnosing and monitoring papilledema (11). Among these abnormalities, the anterior deformation of the pBM is particularly prominent in human patients with ICH and papilledema, when compared to normal individuals or those with other types of disk edema such as pseudopapilledema, non-arteritic anterior ischemic optic neuropathy, and optic neuritis (22). It is supposed that alterations in the translaminar pressure gradient, caused by elevated perioptic subarachnoid space pressure, lead to anterior deformation of the peripapillary sclera and ONH by introducing stress/strain, which modifies the biomechanical properties of load-bearing structures and by exerting direct pressure on the tissues (11, 23–25). Elevated cerebrospinal fluid (CSF) pressure within the intracranial subarachnoid space is propagated along the ONS toward the retrobulbar region. An increase in CSF pressure within the perioptic subarachnoid space inwardly compresses the pia mater and the retrolaminar neural tissue. Due to the Poisson effect, radial compression of the retrolaminar optic nerve may induce elongation in the axial (anterior–posterior) direction, thereby increasing retrolaminar pressure. This elevated retrolaminar pressure exerts an anterior force on the lamina cribrosa. Additionally, the increased CSF pressure directly pushes the peripapillary sclera anteriorly, resulting in globe flattening, clockwise rotation of the peripapillary sclera, and anterior displacement of the lamina cribrosa periphery (23, 24). The degree of anterior deformation depends on the structural geometry (e.g., tissue thickness, radius, and curvature) and material properties (e.g., tissue stiffness) of the ONH and its surrounding neural and connective tissues (23–25).

Funduscopic abnormalities commonly associated with canine optic neuritis, such as a swollen optic disk with blurred margins, congested blood vessels, hemorrhage, and peripapillary retinal detachment, are not apparently distinct from those observed in canine papilledema (5, 20, 26). To the best of the authors’ knowledge, pBM deformation were not found in other forms of optic disk swelling such as optic neuritis and pseudopapilledema (20). Thus, the inward bowing of the pBM observed in the current case represents distinct OCT findings that distinguish papilledema from pseudopapilledema and optic neuritis in dogs, which aligns with a previous study in dogs with experimentally raised ICP (27).

Several chorioretinal changes in papilledema have also been acknowledged, albeit not as widely recognized, including the choroidal neovascular membrane, variable degree of subretinal fluid, macular exudate, and retinal or choroidal folds (11, 28). Subretinal fluid is believed to occur due to the contiguous spread of optic disk edema, leading to the disruption of the ellipsoid zone and resulting in either central or paracentral visual field defects (28). Most cases of subretinal fluid are limited to the peripapillary and macular regions and are difficult to detect clinically using funduscopy, thus emphasizing the need for macular OCT. In contrast, our patient presented with retinal detachments observable over a broad retinal region. Similar lesions have been described as subretinal exudative in optic neuritis and sudden acquired retinal degeneration (29, 30). Wide-ranging retinal detachments observed in the right eye, where ONH edema was not evident, are distinctive features of this case. The differences in the biomechanical properties of the ONH and the peripapillary sclera, as well as variations in the vascular anatomy like the arterial supply and venous drainage system of the eye between humans and dogs, could potentially be contributing factors (31, 32).

To date, there is no widespread agreement on the most effective treatment strategies for sellar masses. Medical management for non-functional sellar mass includes corticosteroids to control brain edema and chemotherapeutic drugs such as hydroxyurea (33, 34). Radiotherapy with various protocols for the treatment of pituitary tumors has been reported, and it is sometimes performed as adjunctive therapy following surgical resection (33–35). Transsphenoidal hypophysectomy is the preferred treatment for humans with sellar masses and is likely to be a treatment of choice in dogs (33, 34). Prognostic factors like tumor size, type, neurological signs, and the extent of expansion into parasellar tissues can significantly affect surgical outcomes (33, 34). However, visual outcomes after surgery for patients with sellar masses and preoperative visual impairments remain unexplored in veterinary medicine. Interestingly, the GCC and RNFL thicknesses have prognostic value for visual field recovery after decompression surgery in humans (36, 37). Likewise, the preservation of GCC and RNFL in the right eye of our patient likely accounts for the positive PLR. However, this preservation may not be sufficient to maintain vision due to a blockage in the conduction of action potentials caused by chiasmal compression or invasion.

## 4 Conclusion

We observed the anterior deformation of ONH and pBM in one eye, while the contralateral eye exhibited signs of retinal detachment. The MRI demonstrated an intracranial mass and neuroimaging indicator of ICH, thus providing compelling evidence for papilledema. Overall, our findings strongly indicate an asymmetric papilledema, especially in the late stages of the disease. It is noteworthy that, to date, sparse reports of papilledema in veterinary medicine have not yielded objective diagnostic criteria. Consequently, we believe our report can offer a more detailed funduscopic and OCT basis for the diagnosis of papilledema in the canine species.

## Data availability statement

The original contributions presented in the study are included in the article/supplementary material, further inquiries can be directed to the corresponding authors.

## Ethics statement

Ethical approval was not required for the studies involving animals in accordance with the local legislation and institutional requirements because all procedures were carried out for diagnostic purposes after obtaining the owner's informed consent. Written informed consent was obtained from the owners for the participation of their animals in this study.

## Author contributions

HC: Writing – review & editing, Writing – original draft, Visualization, Investigation, Data curation, Conceptualization. MC: Writing – original draft, Visualization, Investigation, Data curation. SY: Writing – review & editing, Investigation. MJ: Conceptualization, Writing – review & editing, Supervision. SP: Writing – review & editing, Supervision, Funding acquisition.
